# Deep Vein Thrombosis of the Left Lower Limb in a Sudanese Child with Sickle Cell Disease

**DOI:** 10.3390/medicines9110052

**Published:** 2022-10-22

**Authors:** Alam Eldin Musa Mustafa, Niemat Mohammed Tahir, Nur Allah Elnaji Ahmed Mohamed, Adil Abdullah Mohammed, Sara Ismail Mohammed

**Affiliations:** 1 Department of Child Health, College of Medicine, King Khalid University, P.O. Box 641, Abha 61421, Saudi Arabia; 2 Department of Pediatrics, Faculty of Medicine and Health Sciences, Kordofan University, P.O. Box 160, El Obeid 51111, Sudan; 3 El Obeid Specialized Pediatric Hospital, El Obeid 51111, Sudan; 4 Sudan Ministry of Health, P.O. Box 303, Khartoum 11111, Sudan; 5 Sudan Medical Specialization Board, Khartoum 11111, Sudan

**Keywords:** sickle cell disease, deep vein thrombosis, Sudanese

## Abstract

This is a case of an eleven-year-old female Sudanese child, a known Sickle Cell Anemia (SCA) patient, who presented with fever, as well as left thigh and leg swelling that was associated with pain and warmness, which was diagnosed as Deep Vein Thrombosis (DVT) of her left lower limb. She had a previous history of admissions to the emergency room, during which she once received blood. The patient was managed by carrying out a basic routine initial laboratory investigation. A Doppler ultrasound scan showed features consistent with DVT. Based on the clinical findings and investigation results, management began by providing the patient with intravenous fluid, analgesia, packed Red Blood Cells (RBCs), intravenous antibiotics, and low-molecular-weight heparin. Further consultations showed that there was no need for vascular surgery or surgical intervention. This case highlights the need for more studies on DVT and Venous Thromboembolism (VTE) complications in children with SCA, so as to develop strategies for diagnosis and management in order to reduce the risk of life-threatening complications of VTE in patients with Sickle Cell Disease SCD.

## 1. Introduction

Sickle cell disease (SCD) is a global monogenic disorder with a high incidence rate in many regions due to a lack of awareness among local health policymakers and the public [1]. Therefore, (SCD) remains a global health problem in Sub-Saharan Africa and other regions, although much more scientific progress has been made by many researchers [2]. Most of the prevalence of SCD is in Africa, where little is known about this disease [3]. On the other hand, the prevalence of deep vein thrombosis (DVT) has also increased significantly over the past 21 years in children [1]. DVT cases in the pediatric population are still rare, occurring in about 10 to 14 out of 10,000 pediatric admissions annually [4]. The detection of DVT diagnosis reports requires a multifaceted approach that comprises clinical assessments and the evaluation of pre-test probability [5]. It has previously been reported that 95% of all cases of DVT in children are related to an illness or underlying conditions (congenital heart disease, trauma, cancer, surgery, nephrotic syndrome, inflammatory bowel disease, systemic lupus erythematosus, and especially central venous catheter placement) [6,7]. In children, DVT was reported to lead to many risk factors, including central venous catheters, chronic medical conditions, and thrombophilia; in critical complications, patients will usually present to primary healthcare providers for treatments with pain and swelling of the affected limb [7]. Investigators have reported that patients with SCD are at risk of various thrombotic complications [8,9]. The incidence or number of cases of SCD reported among Sudanese children with DVT is not well-known. This study aims to report a rare case of a female Sudanese child aged 11 years, a known SCD patient, with DVT of her left lower limb, who presented to our emergency room on the 18th of November 2021.

## 2. Case Presentation

An eleven-year-old female with a body weight of 23 kg was admitted to the emergency room of the Al-Obeid Specialized Pediatric Hospital on 18 November 2021.

Her medical history showed that she was diagnosed with sickle cell anemia (SCA) at the age of 9 months in Elkewaiti Hospital via hemoglobin electrophoresis. A review of her medications revealed that she had received blood twice and subscribed to regular follow-ups and regular medication, such as “folic acid and hydroxyl urea”. She had a history of being admitted two times. At 8 years old, due to febrile illness and receiving blood on the 25th of July 2021, she was admitted due to a severe painful crisis, and she received fluids, antibiotics, and analgesia. On 18 November 2021, she was admitted for the third time to the Al-Obeid Specialized Pediatric Hospital, Sudan, with intermittent symptoms of fever for one week and associated sweating, no rigor or convulsion, and a five-day history of swelling in the left thigh and leg. The swelling, which started suddenly, was associated with pain and warmth and interfered with the movement of the affected limb. When reviewing other systems, it was observed that she had no cough, shortness of breath, syncope, or cyanosis, no diarrhea, vomiting, abdominal distention, or yellowish discoloration of the eyes, no change in urine color, amount, or frequency, and no headache, convulsion, or disturbance in level of consciousness. There was no history of recent falls, trauma, or surgery.

**Figure 1:**
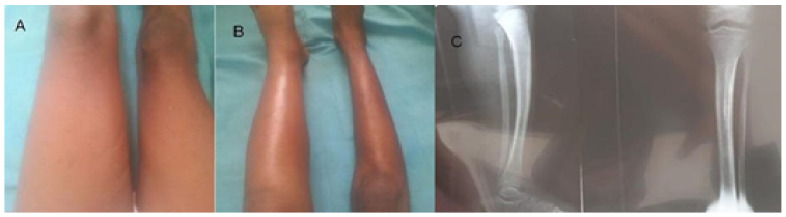
(**A**) Patient on initial presentation, left thigh swelling (mid-thigh circumference 31 cm). Right thigh (mid-thigh circumference 25 cm); (**B**) Patient on initial presentation, left leg swelling (mid-leg circumference 26 cm). right mid-leg circumference 20.5 cm and (**C**) Patient on initial presentation, left and right leg X-ray.

On examination, she was febrile (38c), moderately pale, and not jaundiced; her oxygen saturation was 95% in room air, and her pulse rate, as well as respiratory rate, were normal. Her whole left lower limb was swollen, from her hip to her toe, with a diameter of 31 cm at the thigh and 25 cm at the mid-leg. Additionally, it was warm and tender (Figure 1A,B). The peripheral and femoral pulses were intact. The right limb examination was normal, with a thigh circumference of 26 cm and a mid-leg diameter of 20 cm. The abdominal examination revealed no organomegaly. Furthermore, no abnormalities were found during the cardiovascular and respiratory examinations.

An initial laboratory investigation showed the following:

*CBC: Hb = 4,7 g/dl; MCV = 83; MCH = 28; TWBCs = 21/mm^3^; and platelets = 279,000 c/mm^3^.

*Reticulocyte count: 4, 5.

* CRP: 56; *ESR: 165.

PT: 19,7 *; INR: 1,6 *; and APTT: 28*.

*Renal function, electrolytes, serum calcium, serum magnesium, and liver functions were all determined to be normal.

* Lower limb X-ray: normal (Figure 1C).

**Figure 2:**
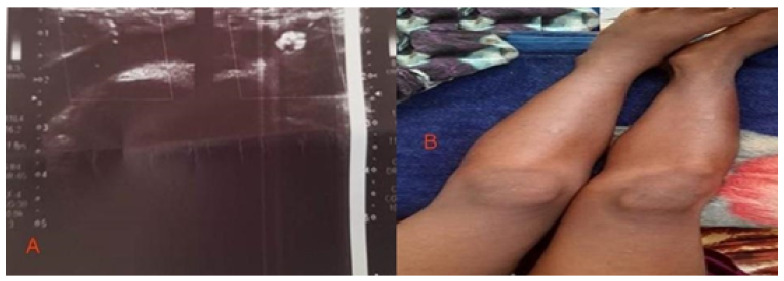
(**A**) Patient on initial presentation, Doppler ultrasound, and (**B**) after one week from management.

*A Doppler ultrasound scan showed that the left femoral vein was not compressible and colorless, and features associated with whole lower limb swelling were consistent with DVT and thrombosis of the common and external iliacs, the common, superficial, and deep femoral veins, as well as the popliteal, anterior, and posterior tibial veins were seen. These extended up to the saphenofemoral and saphenopopliteal junctions (Figure 2A).

## 3. Treatment

Intravenous fluid, analgesia, packed RBCs, intravenous antibiotics, and low-molecular-weight heparin were given to the patient, and a vascular surgery consultation was performed, which revealed that no surgical intervention was required and medical management was advised.

## 4. Outcome and Follow-Up

One week later, the child’s condition improved, the swelling subsided, and the child was able to walk on both limbs (Figure 2B).

## 5. Discussion

The prevalence of SCD in Sudan is found to range from 2 to 30.4%, with a higher prevalence among tribes originating from western Sudan [10,11]. Venous thromboembolism (VTE) is common in adults with sickle cell disease [12]. Pulmonary embolisms have been found to be the most frequent venous thromboembolism, followed by deep vein thrombosis (DVT) [12]. The etiology of increased VTE risk in SCD is multifactorial. These patients have both the common causes of DVT and the specific risk factors for SCD. Frequent hospitalization and immobilization for pain crises and surgeries may contribute as common risk factors [13]. Sickle cell patients have all aspects of Virchow’s triad (venostasis, hypercoagulability, and endothelial injury). In addition, they have increased platelet activation and altered functions as well as abnormalities in the coagulation cascade, which contribute to thrombophilia. Deformed sickle erythrocytes may enhance involvement in clot formation compared to normal red blood cells [12,14]. The VTE risk of certain Hb genotypes over others has been controversial [12].

Patients with SCD have an increased risk of VTE independent of the frequency of hospitalization [15]. Recent evidence demonstrates that there is a high incidence of unprovoked/recurrent venous thromboembolism (VTE) in SCD, with an increased risk of mortality in patients with a history of VTE [14].

DVT is rare in children compared to adult patients [15]. In a multicenter study conducted over seven years [13], only 0.2% of patients with SCD developed VTE. The incidence of DVT was discovered to be 0.02 percent [16]. Children with SCD have an increased risk of DVT when exposed to other factors, such as the placement of a Central Venous Catheter (CVC) [13]. Of pediatric SCA patients who have a CVC inserted, 10% develop DVT [16]. This risk is found to be reduced by using thromboprophylaxis and hydroxyurea in patients with SCD and CVCs [17]. The placement of CVCs in the subclavian vein has been reported to be associated with a much higher risk of DVT compared to them being placed in the internal jugular vein [18]. In contrast to our case, DVT in children is found to be more frequent in the upper limbs than in the lower limbs [19].

Female SCD patients have a greater risk of developing VTE than males do, which is different from the incidence in the general population, where males predominate [13,15]. Other factors that were associated with an increased risk of VTE were the length of hospitalization, Intensive Care Unit (ICU) utilization, and older age [13]. In a study performed on adult SCD patients, 60% of VTE events occurred in less than 90 days of a prior inpatient hospital discharge, with 94% of these having an inpatient admission lasting more than 3 days [20]. This is similar to the situation in our case, where she was admitted less than 60 days before presenting with DVT. The median age in pediatric patients for developing events has been found to be 15.9 (+ or −7.4) years old [20]. Other commonly identified risk factors in children are sepsis, immobility, malignancy, surgery, trauma, and congenital heart disease [15].

The treatment of acute VTE is the urgent use of anticoagulation therapy to prevent complications of fatal pulmonary embolisms and early recurrence, with a 3-month duration of anticoagulant treatment to reduce the risk of recurrence. This treatment is according to the guidelines of management for the general population because of the lack of clinical trials specific to SCD patients [20,21].

## Data Availability

The datasets used and/or analyzed during the current study are available from the corresponding author and study investigators on reasonable request.
